# Sensitization profiles to olive pollen allergens and allergic respiratory disease severity in patients from Jaén, Spain: A cross-sectional study^☆^^☆☆^

**DOI:** 10.1016/j.waojou.2025.101030

**Published:** 2025-02-24

**Authors:** Manuel Alcántara Villar, Sara Anaya Anaya, Alicia López Guerrero, Alba Martínez Chamorro, Carmen Rosa Garrido

**Affiliations:** a Allergology Unit, Jaén University Hospital, Jaén, Spain; b Immunology Unit, Jaén University Hospital, Jaén, Spain; c Support in Methodology and Statistics Area, Fundación para la Investigación Biosanitaria de Andalucía Oriental (FIBAO), Jaén University Hospital, Jaén, Spain

**Keywords:** Asthma, Immunogloblin E, Molecular diagnostic techniques, Ole e I protein, *Olea europaea*, Ole e VII protein, *Olea europaea*, OLE9 protein, *Olea*, Olive pollen, Rhinitis

## Abstract

**Objective:**

To determine the molecular sensitization profile of patients allergic to olive pollen and evaluate its correlation with the type and severity of the allergic respiratory disease (ARD).

**Patients and methods:**

Observational, cross-sectional study including patients aged 5–55 years with seasonal ARD (rhinitis and/or asthma) due to olive pollen sensitization from the Jaén University Hospital (Jaén, Spain), an area with prolonged high olive pollen exposure. Specific IgE (sIgE) levels to Ole e 1, Ole e 7, and Ole e 9 and clinical variables were considered. ARD severity was categorized according to the Allergic Rhinitis and its Impact on Asthma (ARIA) guidelines (rhinitis) and the Guía Española para el Manejo del Asma (GEMAv4.2).

**Results:**

We included 1111 patients (mean [SD] age: 23.4 [12.7] years, 47.7% female) with asthma (87.8%) and rhinitis (96.9%). Ole e 1 sensitization was the most prevalent (79.6%), followed by Ole e 7 (62.0%) and Ole e 9 (50.8%); 60.3% of patients were sensitized to more than 1 olive pollen allergen and 19.2% had negative sIgE results. Ole e 1, Ole e 7, and Ole e 9 sensitizations were associated with asthma diagnosis and severity (*p* < 0.001 for all), Ole e 7 sensitization with rhinitis diagnosis (*p* = 0.006), and Ole e 1 and Ole e 9 with rhinitis severity (*p* = 0.007 and *p* = 0.006, respectively). The Ole e 1, Ole e 7, and Ole e 9 triple sensitization profile was associated with asthma diagnosis (*p* < 0.001) and severity (*p* = 0.029), and with rhinitis severity (*p* = 0.009).

**Conclusion:**

Sensitizations to the olive pollen allergens Ole e 7 and Ole e 9 are prevalent in areas with prolonged high pollen exposure and become major allergens together with Ole e 1. In these areas, a considerable proportion of patients allergic to olive pollen have negative sIgE results. Triple sensitization to Ole e 1, Ole e 7, and Ole e 9 is associated with ARD severity and asthma diagnosis. The sensitization profiles based on molecular diagnosis (MD) may affect decisions regarding allergen immunotherapy treatment.

## Introduction

Allergic respiratory disease (ARD), including allergic rhinitis (AR) and allergic asthma (AA), is mainly caused by seasonal or perennial exposure to pollens, dust mites, molds, or animal dander.^1^ The mechanisms leading to pollen-induced inflammatory and allergic responses are very complex, involving multiple cell types and molecules.^2^ Pollen grains contain proteolytic enzymes that disrupt tight junctions between airway epithelial cells, facilitating allergen delivery.^3^ Moreover, pollen allergens activate Toll-like receptors (TLRs) and pattern recognition receptors (PRRs), triggering immune signaling pathways.^4^ These immune responses consist of mast cell activation and release of vasoactive amines such as histamine, driving the inflammatory cascade typical of allergic reactions.^4^ Moreover, TLRs activation in dendritic cells promote Helper T type 2 cells (Th2) responses, the main regulators of IgE production, further inducing inflammatory responses leading to AR and AA symptoms.^5^

ARD significantly impacts patients' quality of life^6^ and its prevalence is expected to increase due to air pollution and meteorological factors that modify the allergenicity of pollen particles.^7^^,^^8^ In Spain, pollen grains are the leading cause of ARD, with grass pollen being the most frequent sensitization, followed by olive (*Olea europaea*) pollen.^9^^,^^10^ Olive pollen is an important cause of pollinosis in Mediterranean regions with a high density of olive cultivars,^11^^,^^12^ including the South of Spain, where it may become the primary cause of ARD. Moreover, olive pollen is considered highly allergenic and is associated with increased severity of ARD, triggering asthma exacerbations.^13^^,^^14^

The prevalence of patients with polysensitization (ie, sensitized to more than 1 pollen allergen) is high, particularly in regions with higher pollen allergy pressure (i.e., combination of intensity and duration of exposure to an airborne allergen), such as the Mediterranean region.^15^ In the South of Spain, 2 important aeroallergen sources, grass and olive pollen, co-exist and overlap, with the grass pollen season extending from March to August and the olive pollen season from May to June.^9^^,^^16^^,^^17^ Moreover, olive pollen may reach very high counts for prolonged periods.^9^ In this situation, minor allergens that rarely sensitize become major allergens, increasing sensitization prevalence.^18^ For example, in Jaén (Andalucía, Spain), with over 70 million olive trees, sensitization to the minor olive pollen allergens Ole e 7 and Ole e 9 is highly prevalent, together with sensitization to the major olive pollen allergen Ole e 1^19^ In this scenario, the etiologic diagnosis of ARD is challenging, and conventional techniques based on whole extracts, including skin prick test (SPT) and determination of IgE to the whole extract (ImmunoCAP), may be inaccurate.^18^

Molecular diagnosis (MD) by components or component-resolved diagnosis based on the determination of the specific IgE to the main allergens provides an accurate diagnosis.^20^^,^^21^ Besides Ole e 1, several other molecules with an associated biological function have been identified, such as actin-binding protein (the profilin Ole e 2), polcalcin (Ole e 3 and Ole e 8), glucanase (Ole e 9 and its probable degradation product Ole e 4), superoxide dismutase (Ole e 5), and lipid transfer protein (Ole e 7).^22^ MD eliminates the interference of confounding factors, including pan-allergens such as profilins and, to a lesser extent, polcalcin^23^. MD allows allergen immunotherapy (AIT) to be tailored to each patient according to the sensitization profile, potentially increasing its effectiveness and tolerability.^20^^,^^21^ In this regard, sensitization to Ole e7 or Ole e 9 in addition to Ole e 1 has been associated with a higher incidence of adverse reactions to AIT.^24^

MD is emerging as the cornerstone for accurate allergy diagnosis. In the context of complex allergies, such as olive pollen, a deeper understanding of the role of each allergen and the sensitization profiles in clinical manifestations is needed. This cross-sectional study aimed to determine the molecular sensitization profile of patients allergic to olive pollen and evaluate its correlation with the type and severity of the ARD (rhinitis and asthma).

## Material and methods

### Study design and population

This observational, cross-sectional study included patients aged 5–55 years with seasonal allergic respiratory disease (clinically diagnosed rhinitis and/or asthma) due to olive pollen sensitization. Patients diagnosed at Jaén University Hospital (Hospital Universitario de Jaén) by a positive skin prick test (mean diameter >3 mm) and/or positive specific IgE (sIgE), defined as a CAP class 2 or higher (≥0.71 KU/L) to olive pollen, were consecutively included between February 2020 and March 2020. Patients were required to have at least 2 years of disease evolution and a sIgE determination to the major olive allergens (Ole e 1, Ole e 7, and Ole e 9) between January 2014 and December 2019. Patients with incomplete medical records were excluded.

Patients' data were extracted from clinical records and collected in a case report form in an anonymized fashion. Patient informed consent was not required and was deemed unnecessary by the Research Ethics Committee reviewing the protocol. This study followed the General Data Protection Regulation (EU) 2016/679 (GDPR) and Organic Law March 2018 of December 5 on the Protection of Personal Data and guarantee of digital rights in Spain. The study was developed in accordance with ethical principles originating from the latest version of the Helsinki Declaration accepted by local authorities and in line with Good Clinical Practice (GCP) and the requirements of current Spanish regulations. This study was approved by the Research Ethics Committee of the Jaén province.

### Objectives and variables

The main objective of this study was to describe olive pollen sensitization profiles in a population with respiratory seasonal olive pollen allergy from Jaén (Spain) using molecular diagnostic techniques. Secondary objectives were the relationships between the patients' molecular sensitization profile and the type and severity of the ARD (asthma and rhinitis).

sIgE levels to the most relevant olive pollen allergens, Ole e 1, Ole e 7, and Ole e 9, were measured using ImmunoCAP (Thermo Fisher Scientific). For each allergen, sIgE levels <0.35 KU/L were considered a negative result (no sensitization), and levels ≥0.35 KU/L were considered sensitization. The severity of the respiratory allergic disease was categorized based on the guidelines for rhinitis, Allergic Rhinitis and its Impact on Asthma (ARIA) guidelines^25^ and, for asthma, the *Guía Española para el Manejo del Asma* v4.2 (Spanish Asthma Management Guidelines, GEMA).^26^

In addition, this study considered demographic (age and sex) and clinical variables, including patients' clinical diagnosis (rhinitis and/or asthma), family history of allergic disease, other allergic diseases, and time of allergic respiratory disease evolution.

### Statistical analysis

For the sample size calculation, we assumed a ≥15% difference in the proportion of patients with persistent or moderate-severe asthma or rhinitis between those sensitized to olive pollen and those with no sensitization. Considering a 10% dropout rate, a sample of 294 patients (147 sensitized and 147 not sensitized) was deemed necessary to detect differences in the null hypothesis contrast H₀:p1 = p2 using a bilateral Chi-square test for 2 independent samples with an 80% power and a 5% significance. The sample size was calculated using the Ene 3.0 software.

Qualitative variables were presented as frequencies and percentages, quantitative variables as the mean and standard deviation (SD), and the median with the 25th (Q1) and 75th percentiles (Q3). The association between sensitization to Ole e allergens and diagnosis and severity of the ARD (ie, asthma and rhinitis) and the association between the sensitization profiles and the presence of ARD and its severity were analyzed using the Pearson's Chi-square test with Yates' continuity correction and the Fisher's exact test, based on the table distributions. Multiple comparisons were analyzed using the Z-test with Bonferroni correction. For these analyses, asthma severity was classified into 3 categories (intermittent, mild persistent, and moderate-severe persistent) to ensure sufficient representation across categories to calculate the tests. Likewise, rhinitis severity was classified into 2 categories (mild intermittent + mild persistent and moderate-severe persistent). For all analyses, significance was set at a two-sided α < 0.05. All analyses were performed using the statistical software IBM SPSS V21 and R version 4.2.1.

## Results

### Characteristics of the study population

This study included 1111 patients with a mean (SD) age of 23.4 (12.7) years and 6.73 (6.78) years of disease evolution. Patients were evenly distributed between the 2 sexes; one-third were ≤14 years old. Regarding ARD, most patients had asthma (87.8%), which was moderate-severe persistent in 55.4% of the cases, and almost all patients had rhinitis (96.9%), mostly moderate-severe persistent (78.8%) (Table 1). No patients received biological therapies.

**Table 1:** Demographic and clinical characteristics of the study population N = 1111.

Sex, *n (%)*	
Men	581 (52.3)
Women	530 (47.7)
Age (years), *mean (SD)*	23.4 (12.7)
Age group (years), *n (%)*	
≤14	356 (32.0)
>14	755 (68.0)
Evolution time (years), *mean (SD)*	6.73 (6.78)
Family history of allergic disease, *n (%)*	574 (51.7)
Allergic respiratory disease, *n (%)*	
Asthma	975 (87.8)
Rhinitis	1077 (96.9)
Other allergic diseases, *n (%)*	
Food allergy	220 (19.8)
Atopic dermatitis	166 (14.9)
Asthma classification, *n (%)*	
Intermittent	159 (14.3)
Mild persistent	201 (18.1)
Moderate-severe persistent	615 (55.4)
Without asthma	136 (12.2)
Rhinitis classification, *n (%)*	
Mild intermittent	16 (1.4)
Mild persistent	185 (16.7)
Moderate-severe persistent	876 (78.8)
Without rhinitis	34 (3.1)

SD, standard deviation.

### Molecular sensitization profiles to the main olive pollen allergens Ole e 1, Ole e 7, and Ole e 9

Sensitization to Ole e 1 was the most prevalent (79.6%), followed by Ole e 7 (62.0%) and Ole e 9 (50.8%) (Table 2). Patients sensitized to more than 1 allergen were the most frequent (60.3%), particularly triple positive (Ole e 1+, Ole e 7+, and Ole e 9+) (37.6%), and 228 (20.5%) patients showed 1 sensitization, mostly to Ole e 1 (14.6%). Patients sensitized to Ole e 7 and/or Ole e 9 but not to Ole e 1 were rare (6.2%). sIgE results were negative for the 3 allergens in 213 (19.2%) patients.

**Table 2:** Sensitization to Ole e 1, Ole e 7, and Ole e 9 and molecular sensitization profiles of the study population, *n (%)* N = 1111.

**Ole e 1 sensitization**	
Yes	884 (79.6)
No	227 (20.4)
Ole e 1-specific IgE (KU/L), median (Q1, Q3) n = 684	12.15 (2.49, 35.38)
**Ole e 7 sensitization**	
Yes	689 (62.0)
No	422 (38.0)
Ole e 7-specific IgE (KU/L), median (Q1, Q3) n = 567	5.96 (1.17, 34.90)
**Ole e 9 sensitization**	
Yes	564 (50.8)
No	547 (49.2)
Ole e 9-specific IgE (KU/L), median (Q1, Q3) n = 467	19.60 (5.61, 41.4)
**Molecular sensitization profiles**	
Ole e 1+, e 7-, e 9-	162 (14.6)
Ole e 7+, e 1-, e 9-	61 (5.5)
Ole e 9+, e 1-, e 7-	5 (0.4)
Ole e 1+, e 7+, e 9-	135 (12.1)
Ole e 1+, e 9+, e 7-	114 (10.3)
Ole e 7+, e 9+, e 1-	3 (0.3)
Ole e 1+, e 7+, e 9+	418 (37.6)
Ole e 1-, e7 -, e 9-	213 (19.2)

### Association between Ole e 1, Ole e 7, and Ole e 9 sensitizations and ARD type and severity

**Fig. 1:**
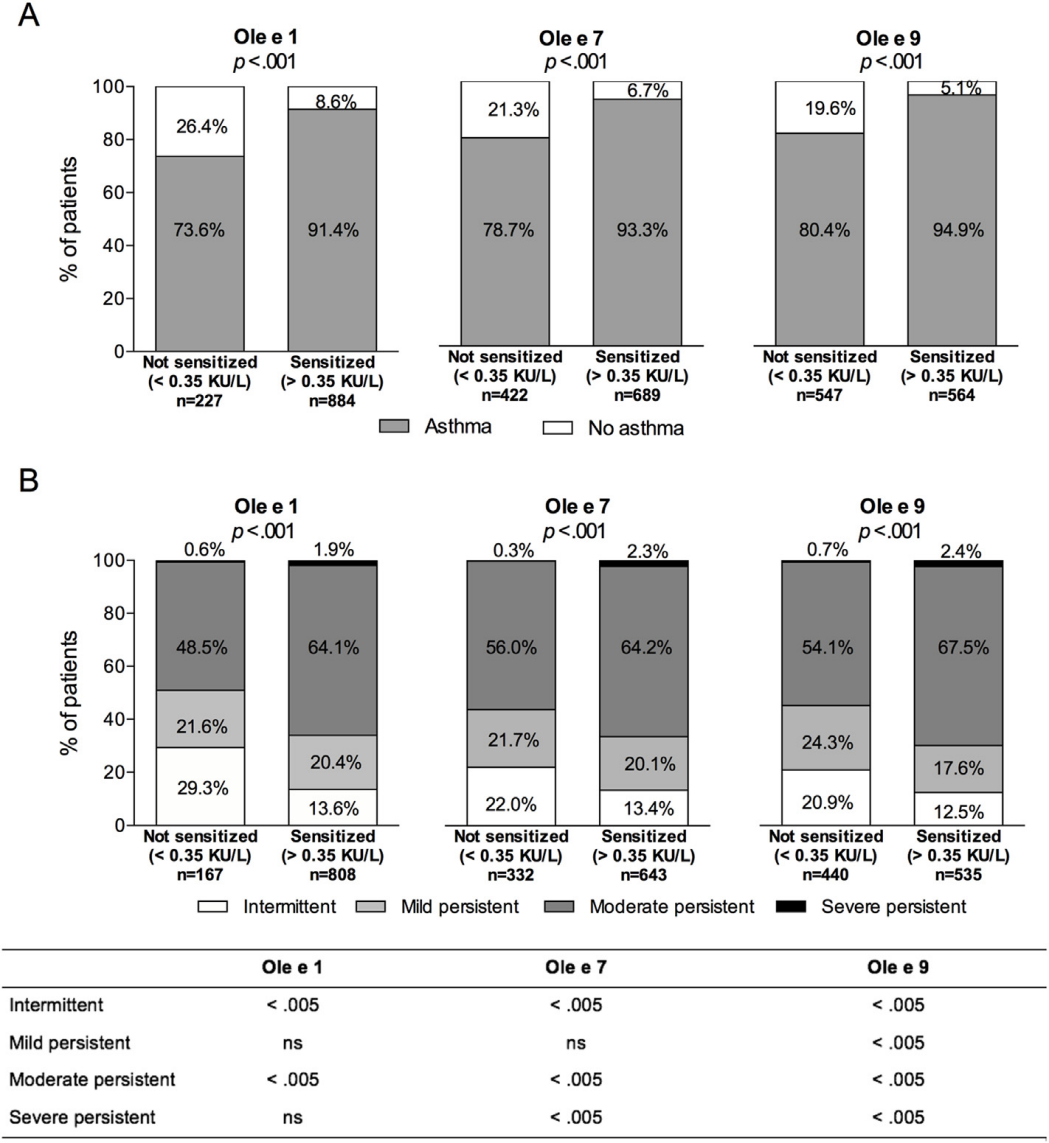
Asthma diagnosis (A) and severity (B) according to Ole e 1, Ole e 7, and Ole e 9 sensitization. P-values for the global comparisons were calculated using the Chi-square test with the Yates' continuity correction in A and the Pearson's Chi-square test in B. P-values for the pairwise comparison in B were calculated using the Z-test with Bonferroni correction.

Sensitizations to Ole e 1, Ole e 7, and Ole e 9 were significantly associated with asthma diagnosis and severity classification (Fig. 1). For olive pollen allergens, a significantly higher proportion of sensitized patients had asthma (*p* < 0.001 for the 3 allergens). The proportion was highest among patients with Ole e 9 sensitization (94.9%) (Fig. 1A). Patients in the more severe asthma categories (i.e., moderate and severe persistent) were more frequent among sensitized patients (Fig. 1B). Pairwise analyses for each severity category showed heterogeneous results for the 3 allergens, with Ole e 9 showing significant differences in all the asthma categories (*p* < 0.005 for all categories).

**Fig. 2:**
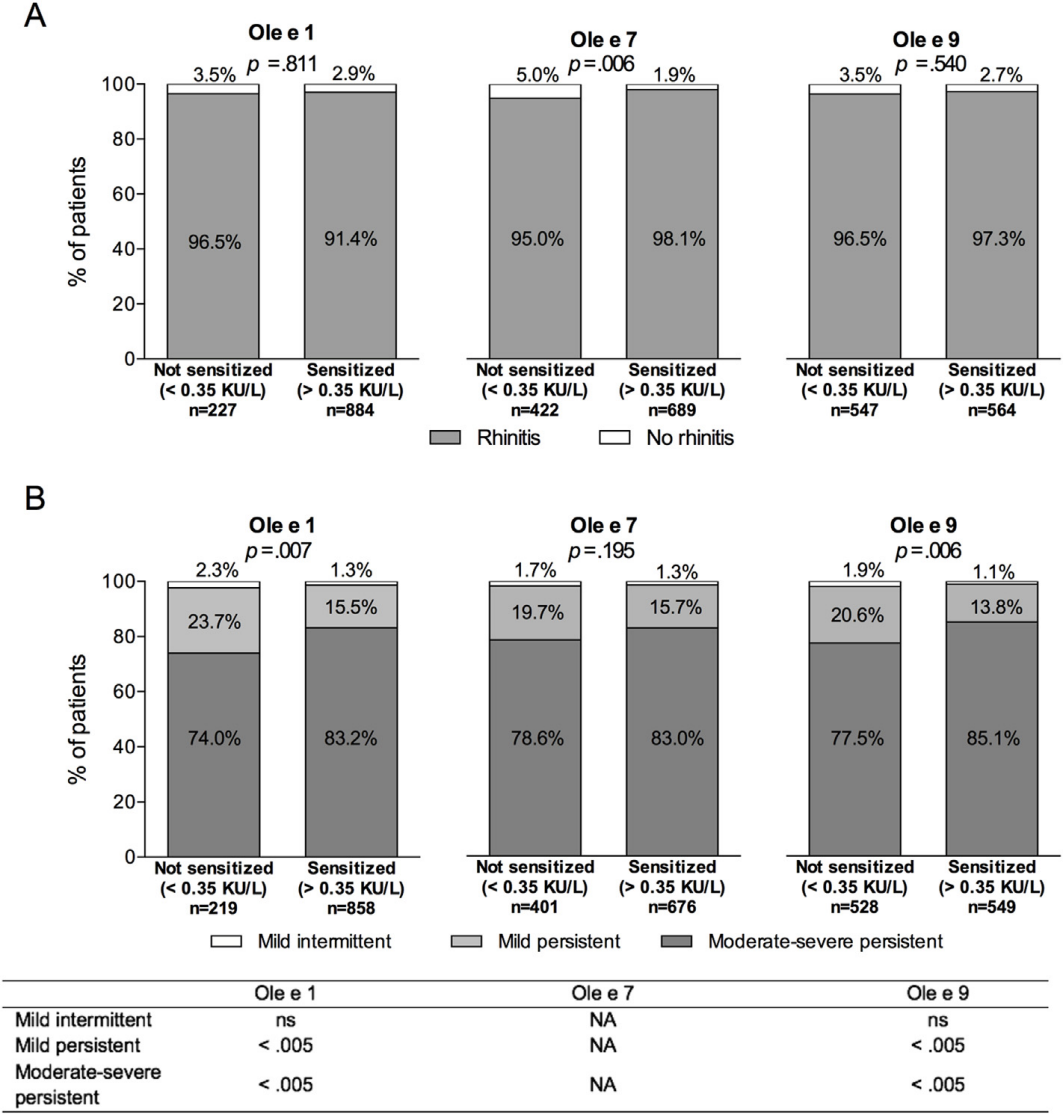
Rhinitis diagnosis (A) and severity (B) according to Ole e 1, Ole e 7, and Ole e 9 sensitization. P-values were calculated using the Chi-square test with the Yates' continuity correction in A and the Pearson's Chi-square test in B. *P*-values for the pairwise comparison in B were calculated using the Z-test with Bonferroni correction. NA, not applicable.

Regarding rhinitis diagnosis, sensitization to Ole e 7 was significantly associated with rhinitis diagnosis (*p* = 0.006), whereas Ole e 1 and Ole e 9 sensitizations lacked significant associations (*p* = 0.811 and *p* = 0.540, respectively) (Fig. 2A). Conversely, rhinitis severity was significantly different according to Ole e 1 and Ole e 9 sensitization (*p* = 0.007 and *p* = 0.006, respectively), with more patients presenting with moderate-severe persistent rhinitis and fewer presenting with mild intermittent and mild persistent (Fig. 2B). Comparisons according to Ole e 7 sensitization did not yield significant differences (*p* = 0.195). Pairwise analyses of severity categories showed significant differences in the proportion of patients with mild persistent and moderate-severe asthma between patients sensitized vs. not sensitized to Ole e 1 and Ole e 9 (*p* < 0.005 for the 2 categories and allergens).

### Association between molecular sensitization profiles and asthma type and severity

**Fig. 3:**
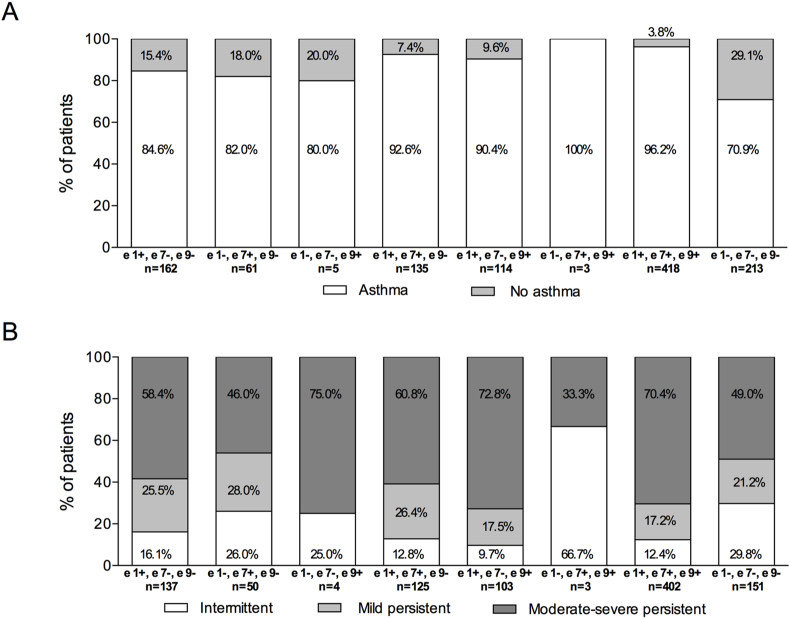
Asthma diagnosis (A) and severity classification (B) according to the sensitization profile to Ole e 1, Ole e 7, and Ole e 9 allergens. Fisher's exact test *p* < 0.001 (A) and *p* = 0.029 (B) for comparisons among sensitization profiles.

The proportions of patients with and without asthma significantly differed according to the molecular sensitization profile to *Olea* allergens (*p* < 0.001) (Fig. 3A). All 3 patients with Ole e 7 and Ole e 9 sensitization and no e 1 sensitization, and 151 (70.9%) of those with no sensitization to Ole e 1, Ole e 7, or Ole e 9, had asthma. Pairwise analyses between sensitization profiles showed significant differences, mostly in comparisons between triple-negative and other profiles (Table 3). Moreover, Ole e 1+, 7+, and 9+ patients were more frequently diagnosed with asthma than Ole e 1+/7- and 9- and Ole e 7+/7- and 9-.

**Table 3:** Multiple comparisons between Ole e sensitization profiles for asthma diagnosis and severity.

	Ole e 1+ Ole e 7 and 9-	Ole e 7+ Ole e 1 and 9-	Ole e 9+ Ole e 1 and 7-	Ole e 1 and 7+ Ole e 9-	Ole e 1 and 9+ Ole e 7-	Ole e 7 and 9+ Ole e 1-	Ole e 1, 7 and 9+	Ole e 1, 7 and 9-
**Diagnosis**								
Ole e 1+/Ole e 7 and 9-		Ns	ns	ns	ns	ns	<0.005	<0.005
Ole e7+/Ole e1 and 9-			ns	ns	ns	ns	<0.005	ns
Ole e9+/Ole e 1 and 7-				ns	ns	ns	ns	ns
Ole e 1 and 7+/Ole e 9-					ns	ns	ns	<0.005
Ole e 1 and 9+/Ole e 7-						ns	ns	<0.005
Ole e 7 and 9+/Ole e 1-							ns	ns
Ole e 1, 7 and 9+								<0.005
Ole e 1, 7 and 9-								
**Severity**								
Ole e 1+/Ole e 7 and 9-		ns	ns	ns	ns	ns	ns	ns
Ole e7+/Ole e1 and 9-			ns	ns	<0.005 ms persistent	ns	<0.005 ms persistent	ns
Ole e9+/Ole e 1 and 7-				ns	ns	ns	ns	ns
Ole e 1 and 7+/Ole e 9-					ns	ns	ns	<0.005 intermittent
Ole e 1 and 9+/Ole e 7-						ns	ns	<0.005 intermittent<0.005 ms persistent
Ole e 7 and 9+/Ole e 1-							ns	ns
Ole e 1, 7 and 9+								<0.005 intermittent<0.005 ms persistent
Ole e 1, 7 and 9-								

ms, moderate-severe; ns, not significant.

P-values were calculated using the z-test with Bonferroni correction.

Asthma severity categories showed significant differences according to the sensitization profile (*p* = 0.029) (Fig. 3B). The percentage of patients with moderate-severe asthma was highest among those with Ole e 9 sensitization but no Ole e 1 and Ole e 7 sensitization (75.0% of Ole e 9+, 1-, and 7-), followed by patients with Ole e 1 and Ole e 9 sensitization but no Ole e 7 sensitization (72.8% of Ole 1+, 9+, 7-) and those with triple sensitization (70.4%). Of patients without sensitizations (ie, Ole e 1-, 7-, and 9-), 49.0% had moderate-severe persistent asthma. Multiple pairwise comparisons between sensitization profiles and severity categories showed significant differences, particularly between triple negative patients and other sensitization profiles in the proportions of intermittent and moderate-severe asthma (Table 3). Compared to Ole e 7+/Ole e 1 and 9- patients, Ole e 1 and 9+/Ole e 7- and Ole e 1, 7, and 9+ patients had significantly more moderate-severe persistent asthma.

### Association between molecular sensitization profiles and rhinitis type and severity

**Fig. 4:**
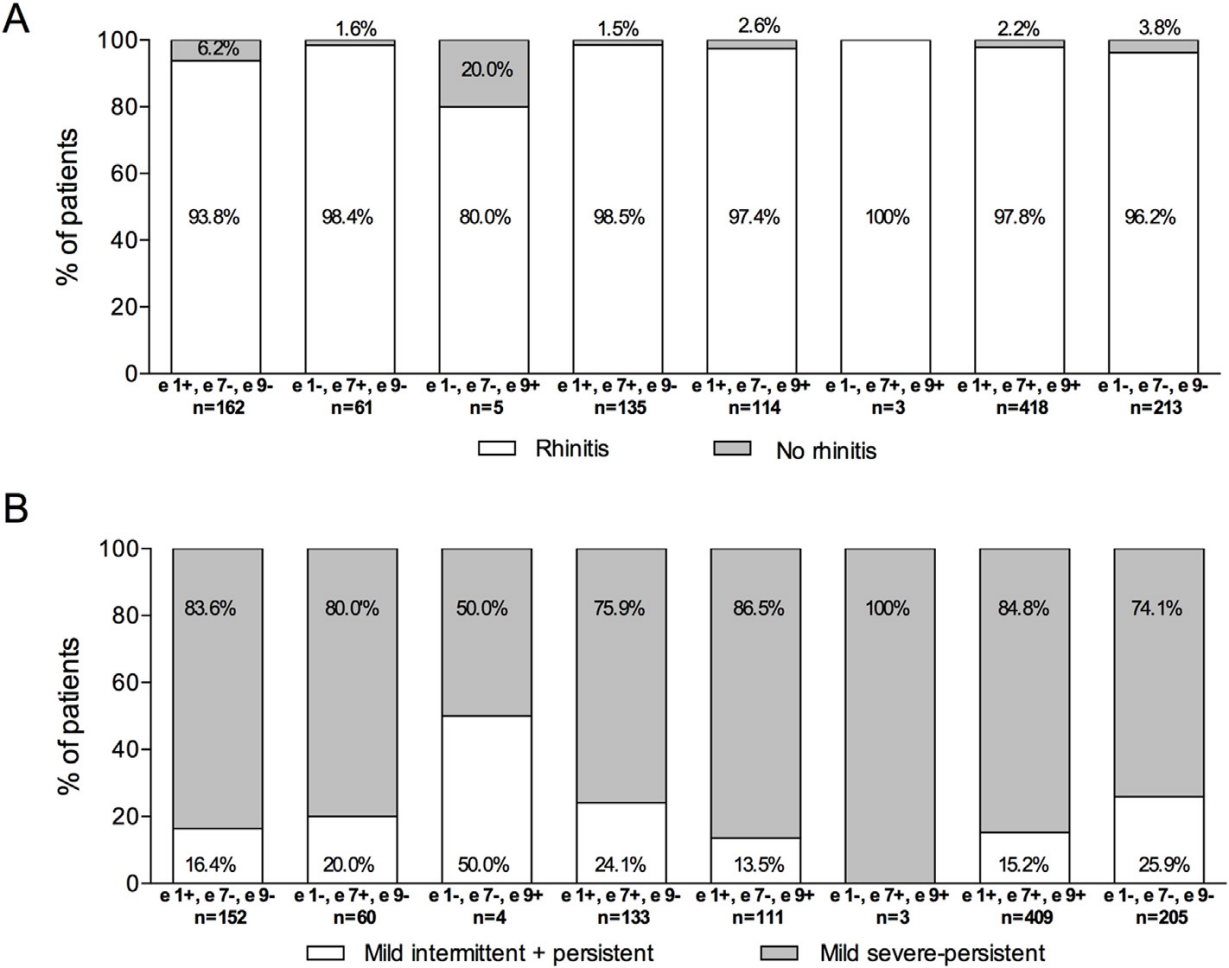
Rhinitis diagnosis (A) and severity classification (B) according to the sensitization profile to Ole e 1, Ole e 7, and Ole e 9 allergens. Fisher's exact test *p* = 0.092 (A) and *p* = 0.009 (B) for comparisons among sensitization profiles.

The percentage of patients with rhinitis did not show statistically significant differences among sensitization profiles, ranging from 80.0% to 100% (*p* = 0.092) (Fig. 4A). Most triple-negative patients had rhinitis (96.2%). However, the percentages of patients according to rhinitis severity significantly differed based on the sensitization profiles to olive pollen allergens (*p* = 0.009) (Fig. 4B). Patients with triple sensitization and those without sensitization had moderate-severe persistent rhinitis in 84.8% and 74.1% of cases, respectively. Multiple pairwise analyses showed that differences between these sensitization profiles were statistically significant for the 2 combined rhinitis categories (Table 4).

**Table 4:** Multiple comparisons between Ole e sensitization profiles for rhinitis diagnosis and severity.

	Ole e 1+ Ole e 7 and 9-	Ole e 7+ Ole e 1 and 9-	Ole e 9+ Ole e 1 and 7-	Ole e 1 and 7+ Ole e 9-	Ole e 1 and 9+ Ole e 7-	Ole e 7 and 9+ Ole e 1-	Ole e 1, 7 and 9+	Ole e 1, 7 and 9-
**Severity**								
Ole e 1+/Ole e 7 and 9-		ns	ns	ns	ns	ns	ns	ns
Ole e7+/Ole e1 and 9-			ns	ns	ns	ns	ns	ns
Ole e9+/Ole e 1 and 7-				ns	ns	ns	ns	ns
Ole e 1 and 7+/Ole e 9-					ns	ns	ns	ns
Ole e 1 and 9+/Ole e 7-						ns	ns	ns
Ole e 7 and 9+/Ole e 1-							ns	ns
Ole e 1, 7 and 9+								<0.005 mild^a^<0.005 ms persistent
Ole e 1, 7 and 9-								

ms, moderate-severe; ns, not significant.

P-values were calculated using the z-test with Bonferroni correction.

a Includes mild intermitent and mild persistent combined.

## Discussion

In this cross-sectional study assessing the sensitization to the main olive pollen allergens (Ole e 1, Ole e 7, and Ole e 9) in a large population of patients with ARD due to olive pollen allergy in Jaén (Spain), an area with extremely high pollen exposure, using MD, Ole e 7 and Ole e 9 sensitizations, considered minor allergens, were highly prevalent (>50% each). Patients with triple sensitization were the most frequent; a substantial proportion had negative sIgE results for the 3 allergens assessed. Ole e 1, Ole e 7, and Ole e 9 were significantly associated with asthma diagnosis and severity. Regarding rhinitis, only sIgE to Ole e 7 was associated with rhinitis diagnosis but not with rhinitis severity, whereas Ole e 1 and Ole e 9 sensitizations were associated with rhinitis severity. Asthma diagnosis and asthma and rhinitis severity differed according to patients' sensitization profiles.

The prevalences of sensitizations to Ole e 7 and Ole e 9 in our study were higher than in other studies in Jaén and Córdoba, reporting the highest prevalence for Ole e 1 sensitization and prevalences of sensitizations to Ole e 7 and Ole e 9 reaching over 35% for both,^15^ 52% and 34%,^19^ and 56.4% and 33.4%,^27^ respectively. Our study provided data from a large sample of patients from an area with extremely high olive pollen counts and showed that Ole e 7 and Ole e 9 behaved as major allergens (Ole e 7, 62.0% and Ole e 9, 50.8%). Consistently, the prevalence of patients with 1 olive pollen sensitization was low. Remarkably, Ole e 7 was the primary sensitizer in 5.5% of patients, similar to previous studies showing a 5.0%–6.0% prevalence.^18^^,^^19^ In this regard, a role for Ole e 7 as a primary sensitizer in areas with high pollen exposure has been suggested.^28^ Regarding Ole e 9 sensitization, except for 8 cases (0.8%), it coincided with Ole e 1 sensitization, as previously reported.^15^ Our data obtained in a large sample further supports the importance, in areas with high pollen exposure, of allergens typically considered minor, where these allergens become major causes of allergy and allergic patients have complex sensitization profiles.

A substantial proportion of patients in our study were sensitized to whole olive pollen extracts but not to Ole e 1, Ole e 7, or Ole e 9 (19.2%), similar to the previously reported 20%.^19^ Among triple negative patients, 70.9% had asthma, which was moderate-severe persistent in half of the cases (49%), and 96.2% had rhinitis, mostly moderate-severe persistent (74.1%). In addition to olive pollen, other aeroallergens may be causing ARD in these patients, but the cause of allergy remains unknown in this study. Future studies focusing on triple-negative patients are needed to investigate the potential role of other olive pollen allergens.

Few studies have assessed the link between sensitization profile to the main olive pollen allergens and clinical manifestations.^27^ In this regard, the allergenic pressure may influence the manifestations and severity of ARD.^1^ Our findings showed robust associations with asthma diagnosis and severity and rhinitis severity, similar to a recent study in Córdoba,^27^ but not with rhinitis diagnosis. In our study, asthma was more severe in Ole e 1, 7, and 9+ and Ole e 1 and 9+/Ole e 7- patients than in Ole e 7+/Ole e 1 and 9- patients, suggesting a role for all 3 allergens in increased ARD severity.

Regarding the associations of single allergens with ARD, previous studies have found that Ole e 7 sensitization is associated with more severe clinical manifestations.^18^^,^^29^ Epidemiologic studies based on MD have shown that only Ole e7 and Ole e 9 were significantly associated with asthma and, consequently, greater disease severity. However, other studies pointed to Ole e 1 as the only allergen related to respiratory symptoms.^15^^,^^30^ In our study, Ole e 7 sensitization resulted in a marginally increased frequency of asthma diagnosis (93.3%) compared to Ole e 1 sensitization (91.4%), with a similar asthma severity. Moreover, Ole e 7 was associated with rhinitis diagnosis but not severity.

In contrast, Ole e 9-sensitized patients showed the highest frequency of asthma, which was moderate and severe persistent at the highest frequencies. These results suggest that Ole e 9 may be associated with more severe forms of ARD. Moreover, our results show robust associations between olive pollen allergens and asthma but not with rhinitis, which supports the notion that olive pollen allergy is associated with more severe manifestations of ARD (i.e., asthma). However, unlike our studies, other studies reported associations of the 3 allergens with rhinitis severity,^27^ whereas our results excluded Ole e 7.

MD is the cornerstone for accurate diagnosis, allowing the identification of the relevant allergens. This information is crucial to prescribe the suitable AIT composition.^31^ However, in the context of complex allergies involving multiple allergens, such as olive pollen allergy, the choice of AIT is more challenging and may require analysis of each allergen content. Immunotherapy products, based on natural extracts of pollens from different sources, contain variable and heterogeneous amounts of the different olive pollen allergens.^32^ This variability may be due, among other reasons, to the intrinsic variability in the amount and potency of olive pollen allergens across cultivars.^33^^,^^34^ Furthermore, the administration regimens and doses have been established based on the effectiveness and safety on averaged populations, likely reacting predominantly to Ole e 1^20^ Results from our study based on MD provide valuable information to tailor AIT treatments to each patient's sensitization profile and therefore, may affect the decision to start AIT, particularly in triple negative patients, and the choice of the AIT. Furthermore, given the previously observed increased risk of systemic reactions associated with Ole e 7 sensitization and Ole e 9^24^ MD may contribute to identifying patients with a higher risk of reactions, optimizing therapeutic decisions. Therefore, these results showing a high prevalence of Ole e 7 and Ole e 9 sensitization support the need to consider each case to improve the effectiveness and safety of AIT approaches. Moreover, 19.2% of patients from our large population with olive pollen allergy were not sensitized to any of the 3 most relevant allergens. This finding underscores the importance of AIT product compositions, which should contain all allergens from the allergen source.

The results from this study should be interpreted in the context of limitations associated with its cross-sectional design, which precluded establishing causative relationships between sensitizations and ARD manifestations. However, this design allowed us to include a large sample of patients and analyze rare sensitization profiles. Moreover, our study used the established sensitization sIgE cut-offs to classify patients as sensitized. However, this arbitrary cut-off is currently under debate, and some studies have shown that a lower cut-off may be clinically relevant.^27^ Future studies are needed to clarify sensitization cut-offs for the main olive pollen allergens. Furthermore, this study retrospectively analyzed data from medical records, resulting in missing information such as sensitization profiles to other aeroallergens. In the era of precision allergology,^35^ understanding the prevalence and clinical implications of sensitizations to specific allergens is fundamental to make AIT treatment decisions. In the cases of olive pollen allergy, given its complexity and association with a severe ARD phenotype with asthma exacerbations, further studies based on MD are needed to determine the sensitization profiles associated with more severe phenotypes and reach a consensus regarding treatment options for these patients.

## Conclusions

Sensitizations to the olive pollen allergens Ole e 7 and Ole e 9 are highly prevalent in a large population of patients with allergic respiratory disease from Jaén, Spain, an area with prolonged high exposure to olive pollen. The main olive pollen allergens, individually and as part of sensitization profiles, were primarily associated with asthma diagnosis and severity and, secondarily, with rhinitis severity. Molecular diagnosis of allergic respiratory disease in patients with a complex respiratory allergy, such as olive pollen allergy, is a valuable tool to identify patients with a high ARD complexity and/or severity and provides valuable information to tailor AIT to patient's sensitization profile.

## Abbreviations

AIT, Allergen immunotherapy; AR, Allergic rhinitis; ARD, Allergic respiratory disease; GCP, Good clinical practice; MD, Molecular diagnosis; SD, Standard deviation; SPT, Skin prick test.

## Authors' contributions

Manuel Alcántara Villar, Sara Anaya Anaya, Alicia López Guerrero, and Alba Martínez Chamorro made substantial contributions to the conception and design of the work, and data acquisition and interpretation. Carmen Rosa Garrido analyzed and interpreted the data. All authors participated in drafting the manuscript and revising it critically for important intellectual content, approved the final version to be published, and agreed to be accountable for all aspects of the work in ensuring that questions related to the accuracy or integrity of any part of the work are appropriately investigated and resolved.

## Funding

This study did not receive funding.

## Declaration of competing interest

Manuel Alcántara Villar, Sara Anaya Anaya, Alicia López Guerrero, Alba Martínez Chamorro and Carmen Rosa Garrido declare that they have no conflicts of interest.

## References

[1] Navarro A.M., Delgado J., Muñoz-Cano R.M., Dordal M.T., Valero A., Quirce S. (2017 May 18). Allergic respiratory disease (ARD), setting forth the basics: proposals of an expert consensus report. Clin Transl Allergy.

[2] Guryanova S.V., Finkina E.I., Melnikova D.N., Bogdanov I.V., Bohle B., Ovchinnikova T.V. (2022 Jun 16). How do pollen allergens sensitize?. Front Mol Biosci.

[3] Runswick S., Mitchell T., Davies P., Robinson C., Garrod D.R. (2007 Nov 1). Pollen proteolytic enzymes degrade tight junctions. Respirology.

[4] Chen K., Xiang Y., Yao X. (2011 Oct 1). The active contribution of Toll-like receptors to allergic airway inflammation. Int Immunopharmacol.

[5] Kubo T., Morita H., Sugita K., Akdis C.A. (First Edition. 2017 Jan 1). Introduction to mechanisms of allergic diseases. Middleton’s Allergy Essentials.

[6] Baiardini I., Braido F., Brandi S., Canonica G.W. (2006 Nov). Allergic diseases and their impact on quality of life. Ann Allergy Asthma Immunol.

[7] Brake D.R., Yaman R.N., Camargo A.R. (2023 Jul 1). Meteorological and environmental factors that impact pollen counts, allergenicity, and thresholds: a scoping review. Allergy Asthma Proc.

[8] Plaza M.P., Alcázar P., Oteros J., Galán C. (2020 Dec 1). Atmospheric pollutants and their association with olive and grass aeroallergen concentrations in Córdoba (Spain). Environ Sci Pollut Res Int.

[9] Delgado Romero J. (2017). Alergológica 2015: epidemiological, clinical and socioeconomic factors of allergic diseases in Spain in 2015. Alergológica 2015: factores epidemiológicos, clínicos y socioeconómicos de las enfermedades alérgicas en España en 2015.

[10] Ojeda P., Sastre J., Olaguibel J., Chivato T. (2018 Jun 25). Alergólogica 2015: a national survey on allergic diseases in the adult Spanish population. J Investig Allergol Clin Immunol.

[11] Liccardi G., D'Amato M., D'Amato G. (1996 Nov). Oleaceae pollinosis: a review. Int Arch Allergy Immunol.

[12] Rojo J., Orlandi F., Pérez-Badia R. (2016 May 1). Modeling olive pollen intensity in the Mediterranean region through analysis of emission sources. Sci Total Environ.

[13] D'Amato G., Cecchi L., Bonini S. (2007 Sep 1). Allergenic pollen and pollen allergy in Europe. Allergy.

[14] Stern J., Pier J., Litonjua A.A. (2020 Feb 4). Asthma epidemiology and risk factors. Semin Immunopathol.

[15] Barber D., Torre FD La, Feo F. (2008 Nov). Understanding patient sensitization profiles in complex pollen areas: a molecular epidemiological study. Allergy.

[16] LETIPharma (2024). https://alergia.leti.com/es/calendarios-polinicos_2052.

[17] Sociedad Española de Alergología e Immunología Clínica (SEAIC) (2024). https://www.polenes.com/es/home.

[18] Barber D., Moreno C., Ledesma A. (2007). Degree of olive pollen exposure and sensitization patterns. Clinical implications. J Investig Allergol Clin Immunol.

[19] Alcántara M., Sáenz de San Pedro B., Cañada C. (2017). Steps towards clarifying the clinical relevance of minor olive allergens in areas with extremely high levels of olive pollen. J Investig Allergol Clin Immunol.

[20] Ansotegui I.J., Melioli G., Canonica G.W. (2020 Nov). IgE allergy diagnostics and other relevant tests in allergy. a World Allergy Organization position paper. World Allergy Organ J.

[21] Benninger M.S., Falcetano G.A. (2024 Apr 1). Molecular allergology and component-resolved diagnosis in current clinical practice. Otolaryngol Clin North Am.

[22] Dramburg S., Hilger C., Santos A.F. (2023 Mar 1). EAACI molecular allergology user's guide 2.0. Pediatr Allergy Immunol.

[23] Hauser M., Roulias A., Ferreira F., Egger M. (2010 Dec 18). Panallergens and their impact on the allergic patient. Allergy Asthma Clin Immunol.

[24] Peñuelas E., Serrano P., Barasona M.J., Saiz V., Fernandez L., Moreno C. (2016 Nov). Sensitization to minor allergens has a direct influence on the outcome of subcutaneous immunotherapy in olive-allergic patients. J Investig Allergol Clin Immunol.

[25] Brożek J.L., Bousquet J., Agache I. (2017 Oct). Allergic rhinitis and its impact on asthma (ARIA) guidelines—2016 revision. J Allergy Clin Immunol.

[26] Comité Ejecutivo de GEMA 4.2 (2016). https://www.semg.es/index.php/consensos-guias-y-protocolos/102-gema-4-2.

[27] Manzanares B., González R., Serrano P. (2023 Oct 4). Back to basics: likelihood ratios for olive and grass pollen specific IgE in seasonal allergic rhinitis. Frontiers in Allergy.

[28] Oeo-Santos C., Navas A., Benedé S. (2020 Apr 1). New insights into the sensitization to nonspecific lipid transfer proteins from pollen and food: New role of allergen Ole e 7. Allergy.

[29] Barber D., Diaz-Perales A., Escribese M.M. (2021 Dec 1). Molecular allergology and its impact in specific allergy diagnosis and therapy. Allergy.

[30] Scala E., Abeni D., Pomponi D. (2016 Feb 1). Ole e 1, Ole e 7, and Ole e 9: Identifying distinct clinical subsets of olive tree-allergic patients. J Allergy Clin Immunol.

[31] Matricardi P.M., Dramburg S., Potapova E., Skevaki C., Renz H. (2019 Mar 1). Molecular diagnosis for allergen immunotherapy. J Allergy Clin Immunol.

[32] Duffort O., Palomares O., Lombardero M. (2006 May 1). Variability of Ole e 9 Allergen in Olive Pollen Extracts: Relevance of Minor Allergens in Immunotherapy Treatments. Int Arch Allergy Immunol.

[33] Alché J.D., Castro A.J., Jiménez-López J.C. (2007). Differential characteristics of olive pollen from different cultivars: biological and clinical implications. J Investig Allergol Clin Immunol.

[34] Celenk S., Vatansever B. (2021 Feb 27). Assessment of heterogeneity of two cultivars of Olea europaea based on the study of their Ole e 1 protein content. Environ Sci Pollut Res Int.

[35] Incorvaia C., Al-Ahmad M., Ansotegui I.J. (2021 Apr 1). Personalized medicine for allergy treatment: allergen immunotherapy still a unique and unmatched model. Allergy.

